# Correction: Level and determinants of pentavalent vaccine dropout during infancy: A hierarchical analysis of community-level longitudinal study

**DOI:** 10.1371/journal.pone.0334536

**Published:** 2025-10-13

**Authors:** Tariku Tesfaye Bekuma, Assefa Seme, Saifuddin Ahmed, Muluemebet Abera

The affiliation for the fourth author is incorrect. Muluemebet Abera is not affiliated with #2 but with #1: Department of Population and Family Health, Institute of Public Health, Jimma University, Ethiopia.
