# Supplementary material for: Level and determinants of pentavalent vaccine dropout during infancy: A hierarchical analysis of community-level longitudinal study
Source: PLoS One. 2025 Jun 26;20(6):e0326684. doi: 10.1371/journal.pone.0326684 (PMC12200679; doi:10.1371/journal.pone.0326684)
Supplement: Attachment — Submitted filename: Comment_plos.docx [file pone.0326684.s001.docx]

**Comment**

1. Add objective to introduction section than a sub-section in abstract?
2. There are a plenty of research o vaccination drop out in Ethiopia with even with spatial and weighted regression analysis, what you adds?
3. Why you don’t use other random effect statistics, median odds ratio, proportional change in variance
4. I am not sure with your community level variables? What are your references cite it?
5. What is the your rational to do spatial analysis, do you think there is a variation across regions? If so how do you know? Have you done auto-correlation?
6. Don’t compare apple to orange, compare apple to apple in your discussion, why your pocket study compared with sub-Saharan African study?
7. Improve the language, consistency and grammar of your entire text?
